# Investigation of thermal conductivity and thermal performance of heat pipes by structurally designed copolymer stabilized ZnO nanofluid

**DOI:** 10.1038/s41598-023-39598-1

**Published:** 2023-08-30

**Authors:** K. S. Pavithra, Vinay Parol, A. Brusly Solomon, M. P. Yashoda

**Affiliations:** 1 Department of Chemistry, Research Centre, GM Institute of Technology, Davanagere, 577006 India; 2 Department of Physics, Bapuji Institute of Technology, Davanagere, 577006 India; 3 Micro and Nano Heat Transfer Laboratory, Department of Mechanical Engineering, Centre for Research in Material Science and Thermal Management, Karunya Institute of Technology and Sciences, Coimbatore, India; 4 Department of Chemistry, Manipal Institute of Technology, Manipal Academy of Higher Education, Manipal, 576104 Karnataka India

**Keywords:** Engineering, Materials science, Mathematics and computing, Nanoscience and technology

## Abstract

The present study concentrated on estimating the thermal conductivity, stability, efficiency, and resistance of a heat pipe for heat exchangers, which were essential for many industrial applications. To achieve this, copolymer of amphiphilic poly (styrene-co-2-Acrylamido-2-methylpropane sulfonic acid) poly (STY-co-AMPS) was synthesized by free radical polymerisation technique. The dispersant were used for homogeneous solution and stabilization of ZnO nanofluids. The effect of dispersant on the thermal conductivity of nanofluids was analysed using a KD2 pro thermal property analyser. There is a significant increase in fluid conductivity had a nonlinear relationship with the volume fraction. The maximum enhancement was observed at an optimized concentration of dispersant at 1.5 vol%. Same time, the influence of dispersant agent on the thermal conductivity of nanofluids were compared with linear polyelectrolytes. Further, the experimental values were compared to the existing classical models based on the reasonable aggrement, the prepared nanofluids were employed as a working medium. The conventional screen mesh heat pipe and the temperature distribution to the thermal resistance of the heat pipe was investigated experimentally. The result shows, optimum concentration of dispersants on nanoparticles exhibits an enhanced heat efficiency as compared with the base fluids. Further, the thermal resistance and temperature distribution show decreased behaviour by increasing the particle volume fraction and dispersant concentration.

## Introduction

From the past two decades, energy storage, energy transmission, and heat generation are the major research domain to which 90% of the global energy budget were allotted. The rapid developments were taken up into advanced thermal materials and technological research. Hence, there is a need to improve the thermo-physical properties of the working fluids which may lead to enhancing the heat transfer performance of the devices. Majorly, engine oil, ethylene glycol and water were widely used as conventional fluids for heat transfer because of their low thermal conductivity, which affects the performance the heat transfer devices. Since the heat pipe is a two-phase heat transfer device which transfers heat with a very low-temperature drop from one place to another. Because of their effective cooling efficiency, these are widely used in various heat transfer applications. The heat pipe is a common cooling system in many thermal applications because the working fluid circulates through a capillary pressure gradient. The phase transition and evaporation/condensation of the working fluid cause substantial variation in heat transfer characteristics The effectiveness of heat pipe is based on the quality/dimension of the material, fluid properties and wick structures^1^. The thermal application includes such as solar systems^2^, gas sensing^3^, electronic cooling^4^, optoelectronics^5^, aerospace^6^, and heat exchangers^7,8^.

For heat transfer applications, nanofluids were used as working fluids which may overcome the drawback of conventional fluids. The stable dispersion of thermal nanofluids is required to fulfil the potential and realize the industrial standards^9^. The main drawback of NPs would be phase-separated and precipitated out of the fluids. During the heat-transfer process, the aggregated nanofluids would cause clogging and abrasion issues in particular microelectronic systems^10^. Achieving dispersion of thermal nanofluids is one of the greatest challenges and many approaches have been attempted which includes the addition of surface charges / utilizing chemical surface modification with surfactants. ultrasonic treatment, mechanical stirring and so on.

Many researchers have been attempted to improve the thermal performances of nanofluids in that, Kang et al.^11^ investigated the effect of silver nanofluids on a sintered heat pipe. According to their findings, there is a decrease in wall temperature input power of 30–50 W. Furthermore, nanofluids as a working medium in heat pipes evidenced higher thermal performance of up to 70 W when compared to water as a base fluid^12^. Similarly, Rosari et al.^13^ investigated the thermal conductivity and heat pipe thermal performance of ZnO-Ethylene glycol-based nanofluids at lower particle volume fractions. In their observation, the temperature distribution and thermal resistance of the heat pipes decreases by increasing the particle volume fraction and crystalline size. Whereas, Jian et al.^14^ reported the thermal performance comparison of oscillating heat pipes (OHP) with SiO_2_ -water and Al_2_O_3_- water nanofluids at a mass concentration of nanoparticles (0–0.6 wt% SiO_2_ and 0–1.2 wt% Al_2_O_3_). It is observed that, the change in surface conditions at the condenser and evaporator is mainly due to different particle, which affect the thermal performance or deterioration of heat pipes^15^. Furthermore, a comparative study of sintered and mesh wick heat pipes on CuO nanofluids was performed by Kumaresan et al.^16^. In their study, the sole effect of sintered wick heat pipes shows enhanced thermal performance and thermal resisitance of heat pipes as compared to the mesh wick heat pipes at 70 W.

Suresh et al.^17^ prepared a hybrid nanofluids Al_2_O_3_–Cu/DI water (90:20 weight proportion) using a dispersant—sodium lauryl sulphate (SLS). Experimental, thermal conductivity and viscosity of hybrid nanofluids were investigated and maximum thermal conductivity was found to be 12.11% at 2% volume concentration. Hamidesh et al.^18^ developed functionalized CNT-water nanofluids by using oxidising agents to improve the cooling capacity of electronic devices. In the thermosyphon, nanofluids with a highly functionalized sodium moiety performed better in terms of higher thermal efficiency and lower thermal resistance. Nanofluids easily get agglomerated due to their high specific surface area, maintaining stability has become a difficult issue^19^. Furthermore, the aggregated nanofluids tend to settled out, which degrades the thermal properties of the nanofluids^20^. In most cases, a high quantity of surfactants needs to be added to get a good stability, resulting in increased thermal conductivity and viscosity. However, the high viscosity of nanofluids needs more power to transport the fluid from one point to another, which hinders the use of these nanofluids in practical applications. To overcome the stability issues, polymer dispersants were used as a stabiliser to improve the long-term stability of the nanofluids. Based on the literature review, less work has been done on polymer dispersants for thermal conductivity and heat pipe experimental techniques.

Recently, functionalized copolymers received significant importance due to their wide range of properties provided by tuning/replacing the structural features such as sulphonyl, carboxylic, amides, aldehydes, anhydrides, soluble polymeric side chains and so on. This creates an effective electrostatic barrier that prevents the agglomeration of the particles and enhances dispersion stability^21^. The surface properties of NPs can be modified by amide functionalized copolymers by changing their solution properties, such as surface charge and colloidal stability^22^.

In the present work, amphiphilic poly (STY-co-AMPS) copolymer as a dispersant was synthesized. AMPS is a hydrophilic sulphonic acid acrylic monomer that is highly reactive and exhibits good thermal stability^23^. The functionalized copolymer has a hydrophobic backbone i.e. Styrene moiety which leads to easy adsorbing onto the cationic surface of the NPs. On the other hand, hydrophilic AMPS moiety creates an electrostatic effect for the proper dispersion of NPs in the fluids. Incorporation of STY and AMPS moieties in the amphiphilic polymer structure is proven to be suitable for the dispersion of NPs into aqueous media. Hydrophilic highly functionlized sulfonyl (–SO_3_), amide (–NH_2_) and hydroxyl (–OH) groups have a high degree of ionization leads to improve the dispersion properties of the NPs. We demonstrate the potential of water-soluble amphiphilic polymers containing –SO_3_ group as dispersants for uniform dispersion of NPs in the fluids. This approach provides a convenient way to stabilize the nanofluids using a modified technique in the presence of structurally designed functional copolymers on the thermo-physical properties of ZnO nanofluids for heat transfer applications.

## Experimental section

### Materials

The chemicals [(AMPS—2-Acrylamido-2-methylpropane sulfonic acid), (AIBA—Azobisisobutyronitrile), (STY—Styrene)] were purchased from sigma Aldrich with 99% purity and styrene was used after the purification process. The solvents (Diethyl ether and Dimethylformamide) were purchased from Merck, India used as received. Double distilled water was used to create the dispersions.

### Methodology

#### Synthesis of Poly (STY-co-AMPS) copolymer

**Figure 1:**
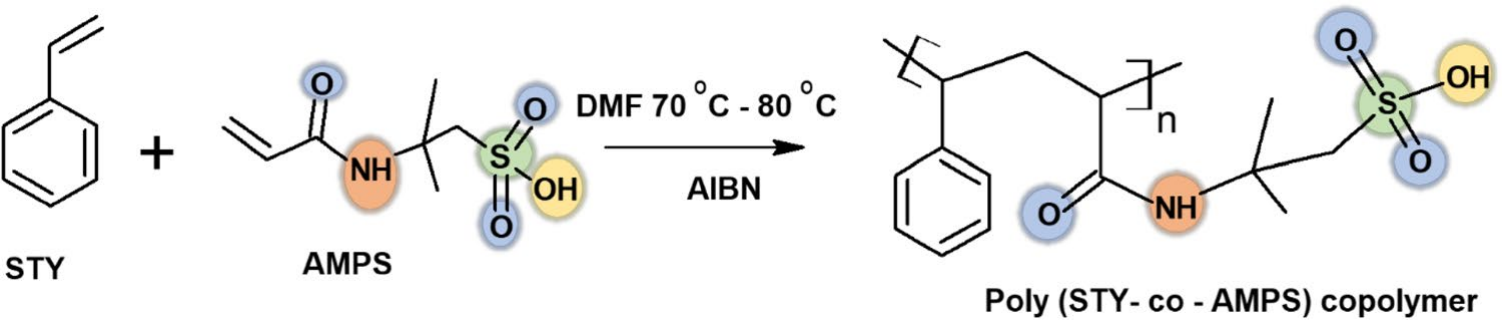
Schematic representation for the synthesis of Poly (STY-co-AMPS) copolymer.

The free radical polymerizations of STY and AMPS are carried out in a typical three-necked round-bottom flask outfitted with a reverse chiller, an N_2_ gas inlet tube, a stirrer, and a thermistor at 70 °C to 80 °C with AIBN as the radical initiator in an N_2_ atmosphere. The mixture is stirred for about 24 h. Further, diethyl ether was used to precipitate the produced copolymer, which was then dried under vacuum at 60 °C to a consistent weight. The scheme of the reaction is represented in Fig. 1. FTIR, ^1^H, and ^13^C NMR spectroscopy were used to determine the structure and compositions of the copolymers.

#### Nanofluids preparation

The synthesis and characterisation of ZnO NPs were described in the previous publication^24,25^. The same ZnO NPs were used in this present study. To ensure even particle dispersion, a determined volume of ZnO NPs was added to the base fluid and sonicated for an hour. The resulting mixture was then ultrasonicated for nearly 2 h at ambient temperature using an ultrasonicator (Make: Dakshin Ultrasonics, India) having a fixed frequency of 22 kHz and output power of 240 W at room temperature for approximately 2 h. The same process was used to create a second batch of nanofluids that contained various STY-co-AMPS copolymer chains of an amphiphilic polymer as a dispersant.

To ensure uniform blending of the dispersant in the base fluids, the different concentrations of dispersant were first dispersed in the base fluid and constant stirring for an hour. Following the dispersant-base fluid mixture, the measured volume concentration of NPs was added. This was followed by the same process as mentioned before. The thermo-physical characteristics of water-based ZnO nanofluids were then examined.

#### ZnO nanofluids: thermal conductivity measurement

KD_2_ Pro ((Decagon Devices, USA) thermal property analyzer was used to evaluate the thermal conductivity of ZnO nanofluids. It includes a 60 mm probe that acts as a heat source as well as a temperature sensor. To operate and carry out the measurements, the probe is attached to a microcontroller. The gadget was calibrated using the glycerine that comes with the equipment to determine the experiment’s accuracy margin.

#### Heat pipes test seup

**Figure 2:**
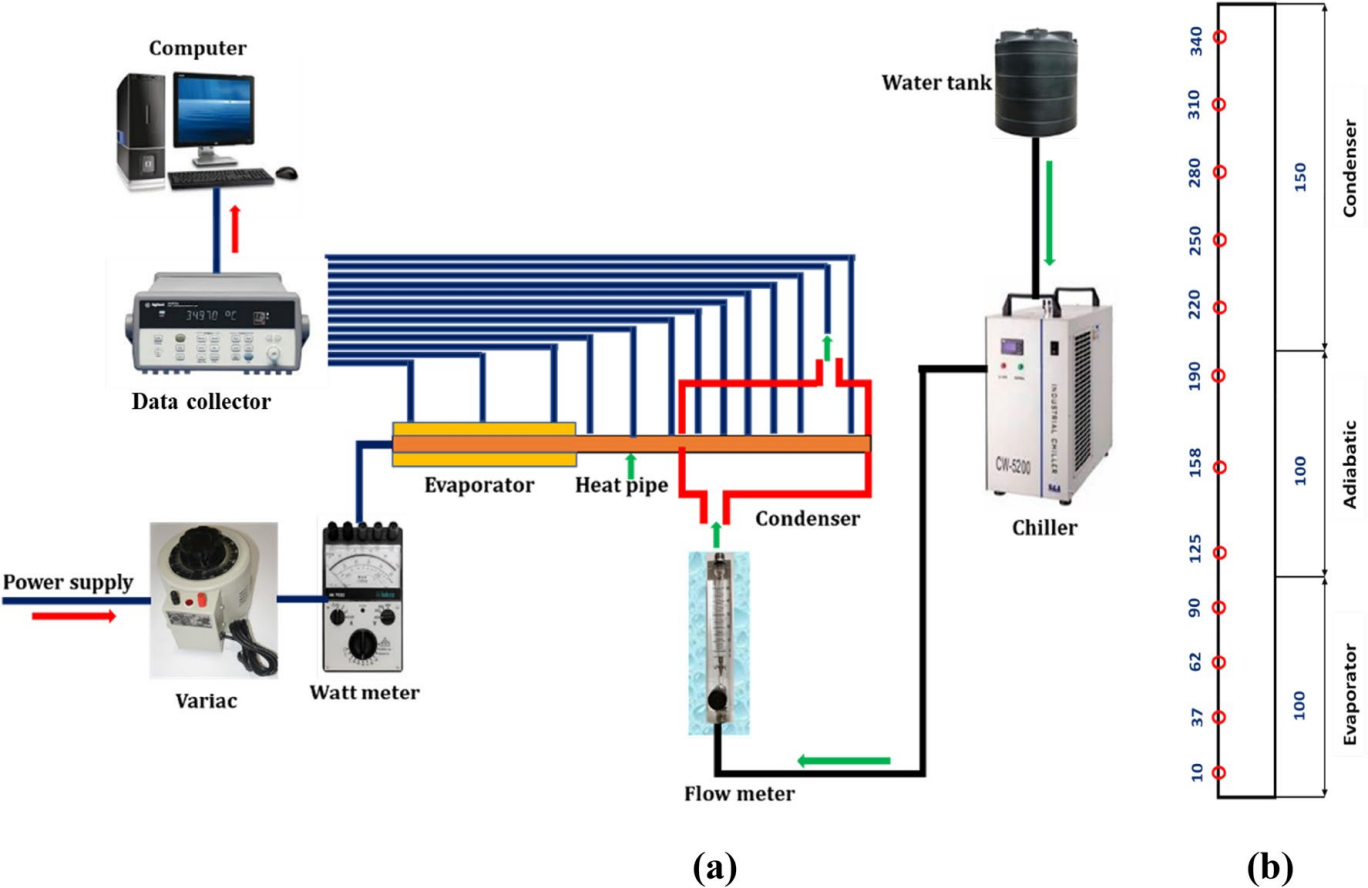
Graphical view (**a**) Experimental setup (**b**) Position of the thermocouple.

As illustrated in Fig. 2, the experimental setup includes a heat pipe, cooling system, data collection, and computer. Heat pipes with an outside diameter of 19.5 mm and a length of 350 mm were designed in order to compare predicted thermal efficiency with experimental evidence. The heat pipe contains four layers of 100 mesh copper wire screen wick (0.009 mm wire diameter). To completely saturate the wick, 12 cc of working fluid is loaded onto the heat pipe after it has been constructed.

In the current experiment, dispersant-stabilised ZnO nanofluids are used as the working fluid. The heater section has a maximum power capability of 1000 W. The evaporator draws its energy from an AC power supply. To prevent thermal resistance between the surfaces of the heater and evaporator, a high thermal conductivity paste is applied. To prevent heat loss between the evaporator and heater surface, a 40 mm thick fibreglass material with a thermal conductivity of 0.04 W/m-K is utilised. The heat pipe’s condenser is made of acrylic material, the chiller delivers a constant flow rate of 320 mL/min of cooling water into the cooling jacket at a constant temperature of 25 ± 0.5 °C.

The flow rate of the cooling water is measured by a flow metre. The cooling water flow rate is found to be 2% uncertain. The temperatures of the heat pipe walls and the cooling water at the condenser’s inlet and outlet are measured with an accuracy of 0.1 °C using OMEGA T-type thermocouples. The heat pipe is positioned horizontally. The heat input used in the experiment’s ranges from 50 to 400 W. Using an Agilent data logger, steady state temperature measurements are made during the experiment for intervals of 30 s. The steady state temperature measurements are then saved in the computer for further processing and date reduction.

### Data reduction

The heat input in the current study is estimated using the voltmeter and ammeter values as follows:

1 $$ Q_{in} = V . I $$

The amount of heat transferred to the coolant water can be calculated from the difference in temperature between the inlet and outlet water, taking into account the water mass flow rate and specific heat as well^26^,

2 $$ Q_{out} = m C_{p} \Delta T $$

where $\Delta T=$(${T}_{out}$ − ${T}_{in}$), ${T}_{out}$ and ${T}_{in}$ are the temperatures of the cooling fluid at its exit and inlet, respectively. m and Cp are the mass flow rate and heat capacity of the coolant, respectively.

Evaporator heat transfer coefficient of the heat pipes^27^ are calculated using Eq. (3)

3 $$ h_{e} = \frac{Q}{{A_{e} \left( {T_{e} - T_{v} } \right)}} $$

In order to evaluate a heat pipe’s thermal performance, it is especially important to consider the temperature drops between the evaporator and condenser. The heat pipe’s overall thermal resistance is described as follows:

4 $$ R = \frac{\Delta T}{{Q_{out} }} $$

where $\Delta T={T}_{e}-{T}_{c}$ , ${T}_{e}$ and ${T}_{c}$ are the average evaporator and condenser wall temperatures respectively. The thermal resistance of the evaporator and condenser are calculated using Eqs. (5) and (6)^28^.

5 $$ R_{e} = \frac{{\Delta T_{e} }}{{Q_{in} }}\quad {\text{where}},\,\Delta T_{e} = T_{e} - T_{vap} $$

6 $$ R_{c} = \frac{{\Delta T_{c} }}{{Q_{out} }}\quad {\text{where}},\,\Delta T_{c} = T_{vap} - T_{c} $$

The average wall temperature in the vapor line was considered as saturation or vapor temperature ${T}_{vap}$.

The thermal efficiency of the heat pipes are calculated by based on the law of thermodynamics^29,30^ using following Eq. (7).

7 $$ \eta_{th} = \frac{{Q_{out} }}{{Q_{in} }} $$

where ${\eta }_{th}-\mathrm{thermal effciency }, \frac{{Q}_{out}}{{Q}_{in}}$—heat out and heat input respectively. The calculate values are tabulated in the Table 1.

**Table 1:** Thermal efficiency of heat pipes.

Q(W)	Thermal efficiency (${\eta }_{th})$		
	Base fluid	ZnO nanofluid	Polymer stabilized nanofluids
50	0.910	1.055	1.025
100	0.898	1.041	1.011
150	0.887	1.030	1.021
200	0.870	1.004	0.994
250	0.871	0.980	0.978
300	0.869	0.941	0.934
350	0.863	0.928	0.914
400	0.849	0.918	0.906

### Uncertainity measurements

Each parameter as measured error. After the above calculations, the uncertainities in the measurement heat transfer coefficient and total thermal resistance were calculated using the following Eqs. (7–10)^27,28^ .

Uncertainity in heat transfer rate is calculated as

8 $$ \frac{\Delta Q}{Q} = \sqrt {\left( {\frac{\Delta m}{m}} \right)^{2} + \left( {\frac{\Delta (\Delta t)}{{\Delta t}}} \right)^{2} } $$

where $\Delta m$—Error in the mass flow rate measurement, $\Delta t$—Error in the temperature difference of cooling fluid and $\frac{\Delta Q}{Q}$ represents the uncertainity present in the estimation of the heat transfer rate.

Uncertainity in heat flux is calculated as

9 $$ \frac{\Delta q}{q} = \sqrt {\left( {\frac{\Delta Q}{Q}} \right)^{2} + \left( {\frac{\Delta A}{A}} \right)^{2} } $$

Uncertainity in the thermal resistance is calculated by

10 $$ \frac{\Delta R}{R} = \sqrt {\left( {\frac{\Delta Q}{Q}} \right)^{2} + \left( {\frac{{\Delta \left( {\Delta T_{hp} } \right)}}{\Delta T}} \right)^{2} } $$

where the ${\Delta T}_{hp}$ and $\frac{\Delta R}{R}$ are the error in the temperature difference and uncertainity in the thermal resistance of the heat pipes respectively.

Uncertainity in heat transfer coeffiecient is calculated as

11 $$ \frac{\Delta h}{h} = \sqrt {\left( {\frac{\Delta q}{q}} \right)^{2} + \left( {\frac{{\Delta \left( {\Delta T} \right)}}{{\Delta T_{v} }}} \right)^{2} } $$

The uncertainties in the measurement of heat flux, thermal resistance and measurement are presented in Tables 2 and 3. It is seen that the uncertainities in the measurements of heat flux, total resisitance and heat transfer coefficients as the heat input increases from 50 to 400 W.

**Table 2:** Uncertainties in the measurement: ZnO nanofluids.

Q(W)	$\Delta Q/Q$	$\Delta q/q$	$\Delta R/R$	$\Delta h/h$
50	1.036	2.467	2.490	3.98
100	1.107	2.627	1.777	3.45
150	1.061	2.454	1.522	2.08
200	1.056	2.451	1.389	2.03
250	1.066	2.487	1.228	1.83
300	1.120	2.690	1.139	1.71
350	1.078	2.527	1.045	1.62
400	1.068	2.488	0.972	1.53

**Table 3:** Uncertainties in the measurement: Polymer stabilized ZnO nanofluids.

Q(W)	$\Delta Q/Q$	$\Delta q/q$	$\Delta R/R$	$\Delta h/h$
50	1.526	3.756	4.349	5.19
100	1.286	3.725	4.192	5.09
150	1.355	3.509	3.681	4.47
200	1.369	3.767	3.589	4.93
250	1.342	3.544	3.603	4.88
300	1.255	3.617	3.890	4.84
350	1.324	3.710	3.606	4.75
400	1.351	3.865	3.518	4.55

## Theoretical models

For the comparison of the effective thermal conductivity of the nanofluids, numerous mathematical models have been brought forth^31^^,^^32^. To calculate the thermal conductivity of ZnO-nanofluids, we used the Hamilton Crosser, Pak and Cho, and Timofeeva models (Eqs. 13, 14, and 15), and we compared the results to experimental observations. ZnO NPs are discovered to have a thermal conductivity of 23.413 W/m-K.

According to the Hamilton and Crosser (H–C) model (1962)^33^^,^^34^ created to determine a two-phase mixture’s effective thermal conductivity,

12 $$ \frac{{k_{nf} }}{{k_{bf} }} = \frac{{k_{p} + \left( {n - 1} \right)k_{bf} + \left( {n - 1} \right)\left( {k_{bf} - k_{p} } \right)\varphi }}{{k_{p} + \left( {n - 1} \right)k_{bf} - \varphi \left( {k_{bf} - k_{p} } \right)}} $$

where ${\mathrm{k}}_{\mathrm{p}}$—Thermal conductvity of the NPs, $\mathrm{\varphi }$—NPs volume fraction, ${\mathrm{k}}_{\mathrm{bf}}$—Thermal conductvity of base fluids, sphericity is defined as the ratio of the surface area of a sphere with a volume equal to that of the particle to the surface area of the particle, and n is the empirical shape factor determined by n = 3/$\varphi$. The H–C model estimates the thermal conductivity ratio, k nf/k bf, for values of ranging from 0.5 to 1.5.

For Al_2_O_3_ and TiO_2_ nanofluids, Pak and Cho^35^ created a thermal conductivity model, which was written as

13 $$ k_{nf} = k_{bf} \left( {1 + 7.47} \right) \varphi $$

The effective medium hypothesis was proposed by Timofeeva et al.^36^ to determine the thermal conductivity ratio for strongly conducting spherical particles. The model makes the unrealistic assumption that the particles are stationary.

14 $$ k_{nf} = k_{bf} \left( {1 + 3\varphi } \right) $$

## Results and discussion

**Figure 3:**
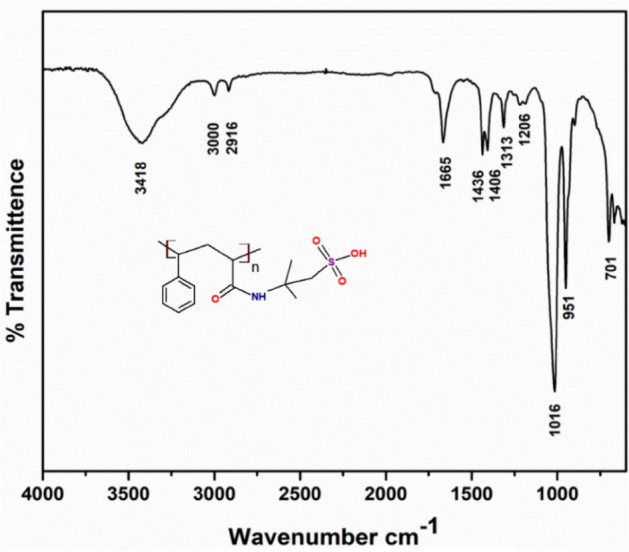
FTIR spectrum for poly (STY-co-AMPS) copolymer.

**Figure 4:**
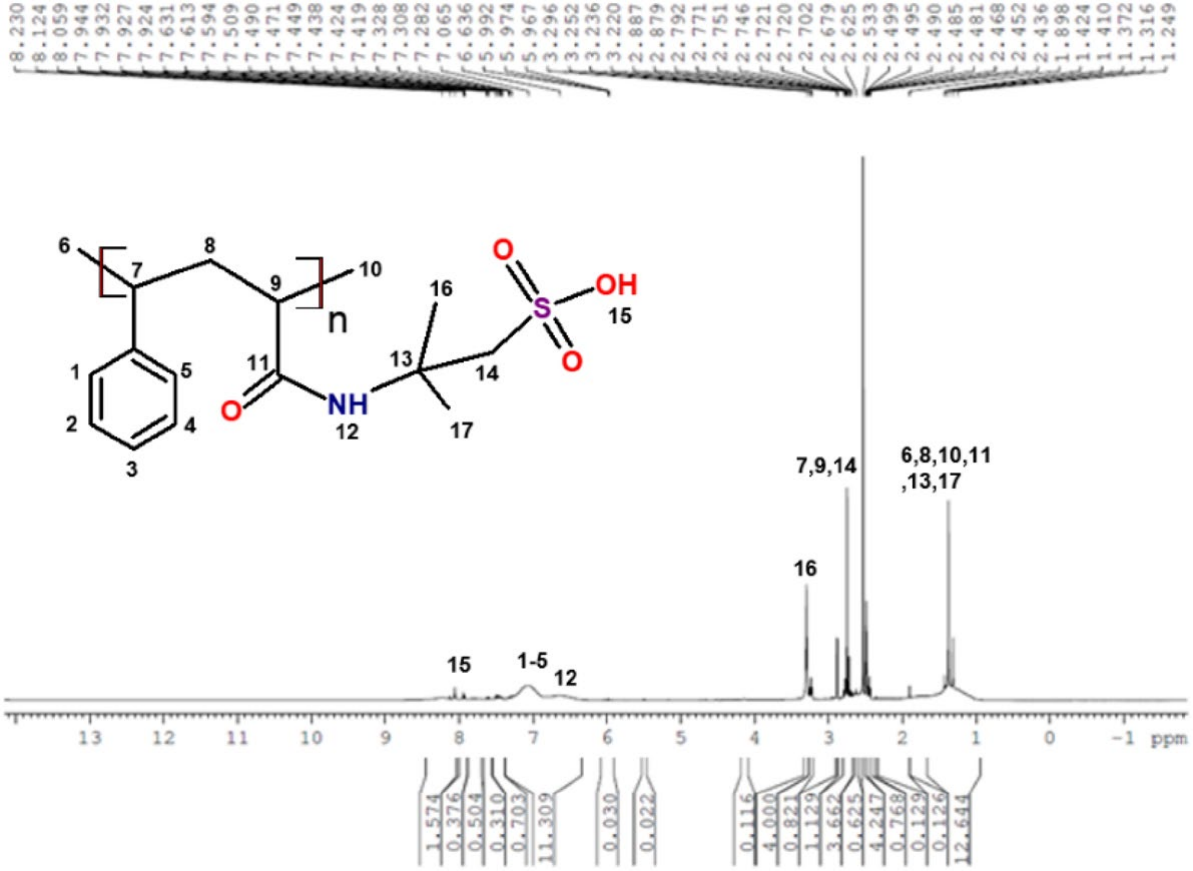
^1^H NMR spectrum for poly (STY-co-AMPS) copolymer.

**Figure 5:**
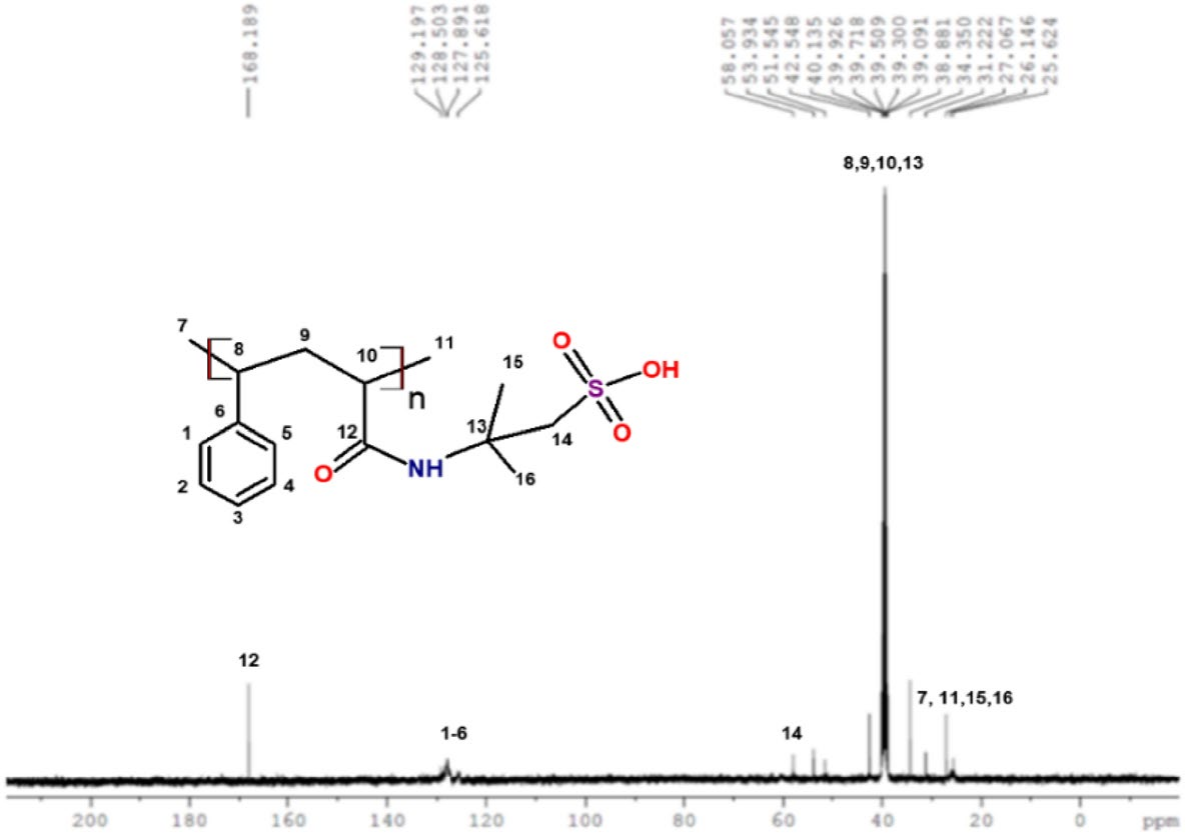
^13^C NMR spectrum for poly (STY-co-AMPS) copolymer.

In Fig. 3. FTIR for poly (STY-co-AMPS) copolymer: Characteristic peaks at 2919–3000 cm^−1^ (Ar –CH of benzene ring), 1436 and 1406 cm^–1^(vibration of –C–C bonds in the benzene ring), 3418 and 1665 cm^–1^ (–NH stretching and C=O stretching), 1406 cm^–1^(–C–N stretching) and 1016 cm^–1^ (stretching vibration of SO_3_H). In Fig. 4. ^1^H NMR (400 MHz, DMSO): *δ* = 2.32–2.56 (–CH and –CH_2_), 7.05–7.96 (Ar –CH), 1.42 (CH_3_ in side chain –C(CH_3_)_2_), 1.25–1.89 (–CH and –CH_2_ for AMPS), 2.68 (–CH_2_–SO_3_H), 6.15–6.78 (broad, NH amide) and 8.22 (–OH) for AMPS. ^13^C NMR (400 MHz, DMSO): *δ* = 125.61–129.19 (p-, o-, m-, Ar –CH), 168.18 (C=O anhydride and amide, overlapping), 51.54–53.93 (C–(CH_3_)_2_), 25.62–42.54 (–CH–CH_2_), 58.05 (CH_2_–SO_3_H) are respective signals confirms the formation of copolymer it is shown in Fig. 5.

**Figure 6:**
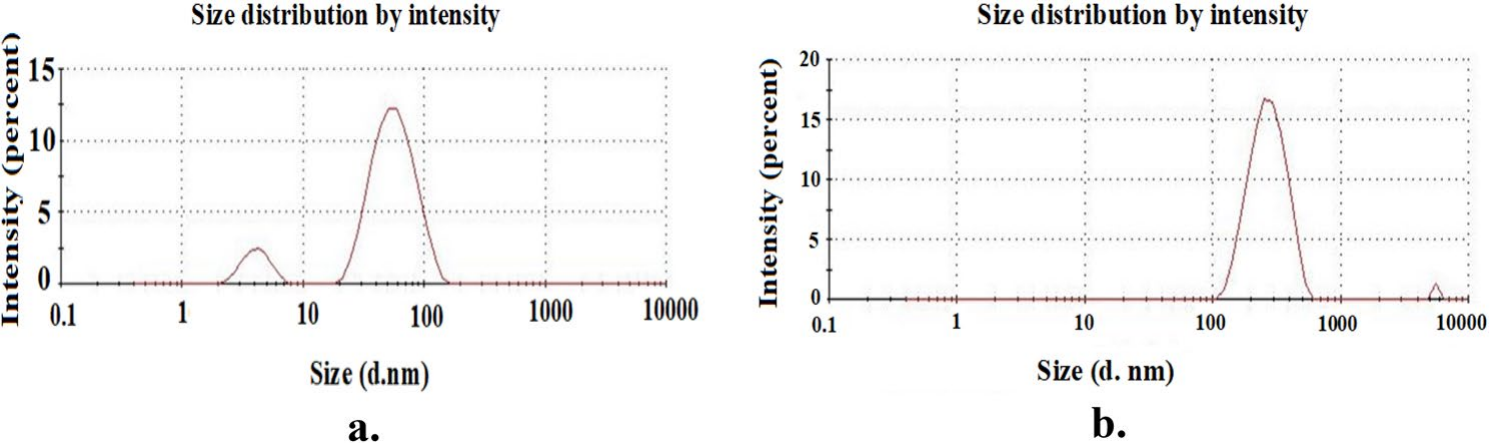
DLS images for (**a**) ZnO nanofluid (**b**) Dispersant stabilized ZnO nanofluid.

A quantitative assessment of the particle size distribution was obtained using DLS analysis. A freshly prepared, diluted sample was used for the analysis, which was carried out at 25 °C. Figure 6a. depicts the ZnO nanofluid’s particle size distribution. The polydispersity index (ratio of the mass-weighted average particle diameter to the number-weighted average particle diameter), as determined by DLS analysis, is 1.001, and the number-averaged particle size is 95 nm. The observed particle size is significantly bigger than the desired nanoparticle size (50 nm), suggesting that thermodynamically stable clusters of particles may have formed. This is primarily due to the adsorbed layer of amphiphilic copolymer dispersants on the cationic surface of the nanofluids, which results in larger particle diameter. Figure 6b. displays the poly (STY-co-AMPS) stabilised ZnO nanofluid particle size distribution, which is found to be 221 nm, and the polydispersity index, which is 0.364.

The polydispersity index value less than one indicates better homogeneity and less clustering. It implies that the dispersants played an active role in the dispersion of ZnO nanofluids due to electrostatic hindrance produced between the copolymer’s adsorbed layer and the NPs in the suspension. It is further confirmed using zetapotential data.

**Figure 7:**
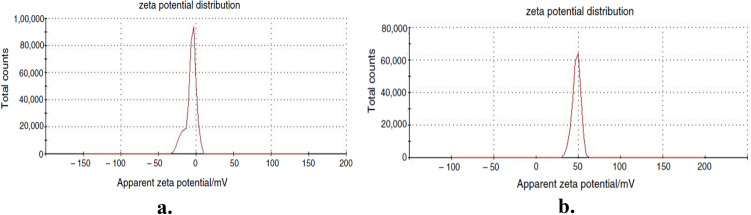
Zeta potential images for (**a**) ZnO nanofluid (**b**) Dispersant stabilized ZnO nanofluid.

The zeta potential was used to assess the stability of NPs dispersion in the base fluids shown in Fig. 7. depicts the zeta potential value of dispersant stabilised ZnO nanofluids with and without dispersant. The zeta potential value for ZnO nanofluids in the absence of dispersant was − 6.78 mV in Fig. 7a, and the potential value slightly shifted from negative to positive with the addition of dispersant, i.e. 50.1 mV in Fig. 7b. It is because an adsorbed layer of poly (STY-co-AMPS) copolymer dispersants on the surface of ZnO NPs creates an effective electrostatic barrier between the particles, ensuring greater stability and dispersibility^37^.

**Figure 8:**
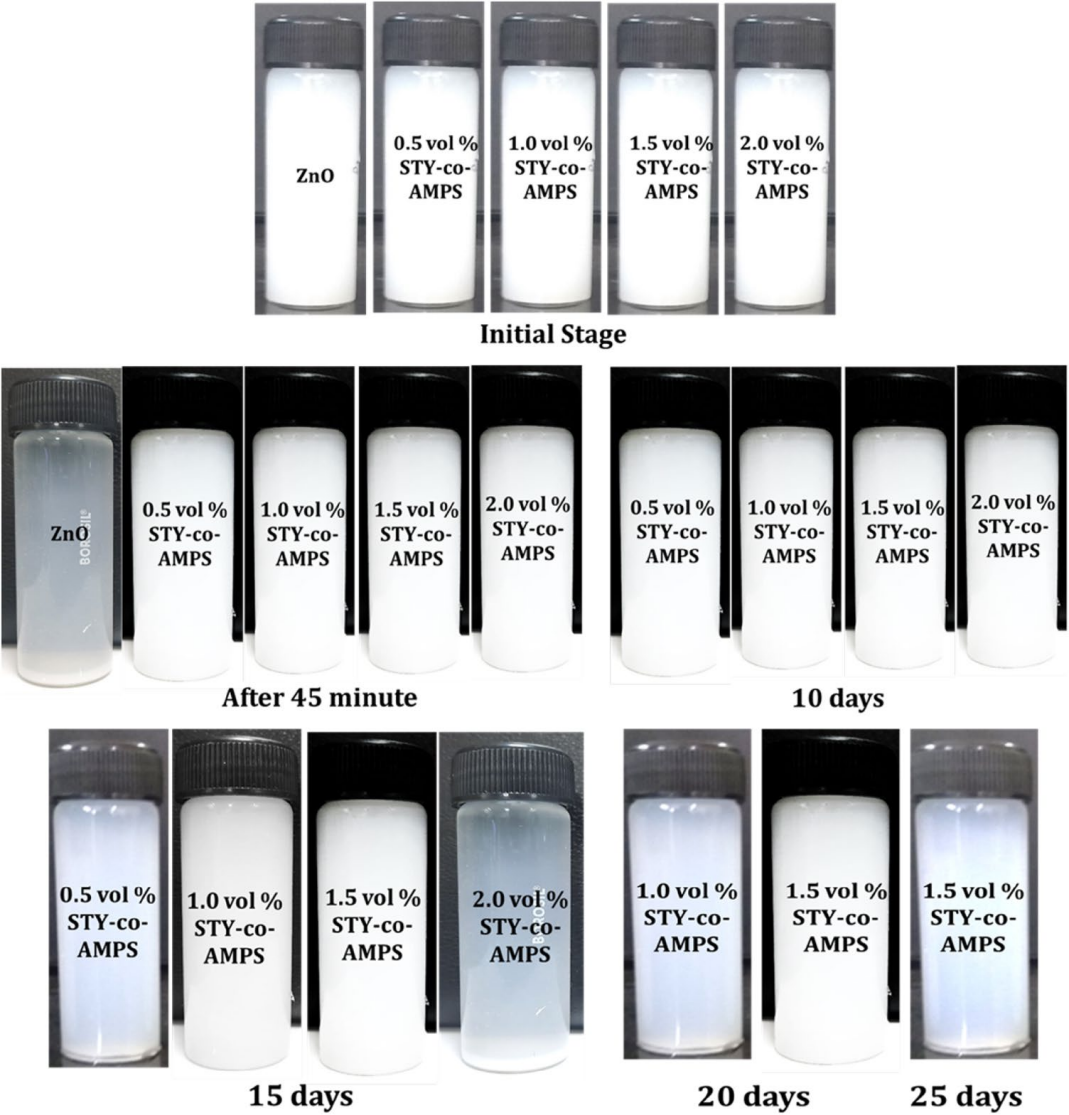
Sedimentation Photographs of with and without dispersant stabilized ZnO nanofluids.

Figure 8 shows photographs of 1 wt% ZnO nanofluids without and with (STY-co-AMPS) copolymer as dispersant at various concentrations ranging from 0.5 to 2.0 vol%. Due to the strong attraction between the NPs, ZnO nanofluids begin to aggregate and settle at the bottom of the vial after 45 min of preparation. The dispersant-stabilised ZnO nanofluids are stable for 10 days at all concentrations. Furthermore, polymer stabilised nanofluids begin phase separation after 15 days of preparation and complete sedimentation is observed after 20 days, with the exception of 1.5 vol% dispersant stabilised nanofluids, which is stable for nearly one month due to sufficient adsorbed molecules creating a strong electrostatic repulsion between NPs and is considered optimal concentration. While the dispersant concentration increased to 2.0 vol%, the stability of nanofluids decreased due to an increase in the adsorbed molecules in the solution, which causes flocculation^38^.

**Figure 9:**
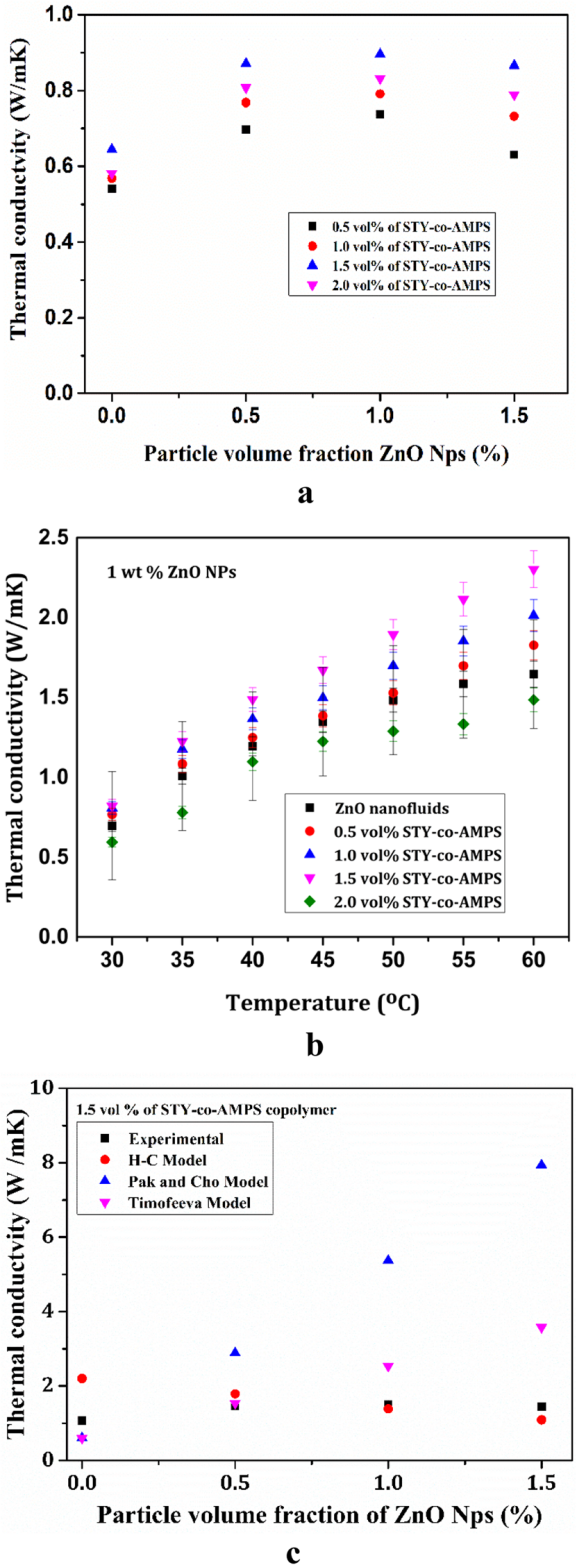
Thermal conductivity of ZnO nanofluids (**a**) At various volume fractions (**b**), different temperatures along with error bars (**c**) Comparison between experimental and a theoretical model.

The thermal conductivity of ZnO nanofluids at a various volume fractions of dispersant and NPs are represented in Fig. 9. The best dispersion and thermal conductivity enhancement of nanofluids are observed at 1.0 vol% ZnO NPs and 1.5 vol% STY-co-AMPS copolymer as a dispersant due to proper dispersion leads to more heat transfer. At 0.931 W/m-K, 1 wt% ZnO nanofluids stabilised by 1.5 vol% dispersant offered the highest thermal conductivity enhancement as shown Fig. 9a.

Figure 9b depicts the investigation into the effect of temperature on the thermal conductivity of ZnO nanofluids, with error bars on the graph indicating the standard deviation over subsequent measurements. Thermal conductivity increases with increasing temperature and gradually decreases with increasing polymer dispersant concentration due to overadsorption of polymer dispersants, which inhibits heat transfer as well as Brownian motion of NPs. The ionic interaction of the highly functionalized hydrophobic styrene moiety adsorbed on the cationic surface of the ZnO NPs and the hydrophilic ionizable sulfonal (${\mathrm{SO}}_{3}^{-}$) and hydroxyl (–OH) functionlized AMPS moiety extended into the fluids suspension creates an effective electrostatic barrier between the NPs in the base fluids, promoting homogeneous dispersion and stabilisation which inturn enhaces the heat transfer^24^^,^^38^.

Figure 9c provides a comparison between the theoretical and experimental thermal conductivity values of ZnO nanofluids. The results revealed that the experimental thermal conductivities are having a slight agreement with the Hamilton and Crosser model and Timofeeva model at lower particle volume fraction (0.5%). However, the Pak and Cho Model over predicts the experimental results.

The uncertainty measurement of thermal conductivity of nanofluids experimental results was determined using the instrument and precision error caused by the deviation in the experimental dataset. KD2 Pro has a 5% instrument error in measuring thermal conductivity^39^. The weighing scale has a precision of 0.01 g. The following yields the uncertainty of the experiment data^40^ .

15 $$\upmu = \pm \sqrt {\left( {\mu_{B} } \right)^{2} + (\mu_{p} )^{2} } \quad {\text{with}}\,\mu_{p} = \pm t _{v, p} SD $$

where ${\mu }_{B}, {\mu }_{p}, {t }_{v, p}$ and SD are bias error, precision or random error in the measurement with p% probability, weighing function $\vartheta$ degree of freedom and sample standard deviation respectively.

16 $$ u_{TCR} = \left[ {\left( {\frac{{\delta k_{eff} }}{{k_{f} }}} \right)^{2} + \left( {\frac{{ - k_{eff} }}{{\left[ {k_{f} } \right]^{2} }} \delta k_{f} } \right)^{2} } \right]^{0.5} $$

where k_eff_ and k_f_ represent uncertainty at *p*% probability, including both instrument and precision errors associated with the measured effective thermal conductivity and thermal conductivity of nanofluids, respectively. Finally, the uncertainty in effective thermal conductivity and TCR (thermal conductivity ratio) was discovered to be between 0.5 and 4.5%.

**Figure 10:**
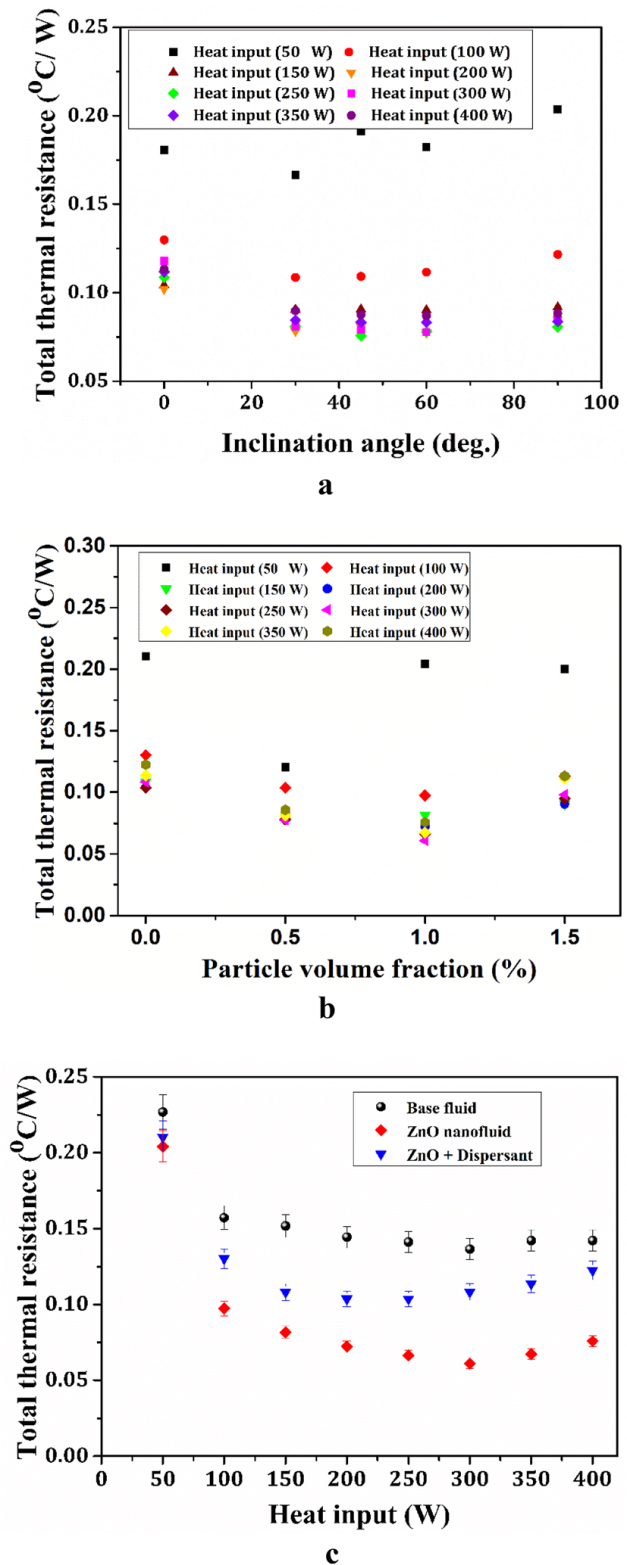
(**a**) Optimization of inclination angle for base fluids (**b**) Total thermal resistance v/s particle volume fraction at various heat inputs (**c**) Variation of total thermal resistance with various heat inputs.

In Fig. 10. Based on the thermal conductivity measurements, the formulated nanofluid was charged into the heat pipe and its performance was evaluated. The overall thermal resistance of the heat pipes between the 0° and 90° angles at various heat inputs (50–400 W) was measured to estimate the effect of inclination angle on the thermal performance of the heat pipe using water as the base fluid, as shown in Fig. 10a. The lowest thermal resistance of the base fluid was observed at 45° inclination angle, which is considered as a base inclination angle for the total thermal resistance of the ZnO nanofluids for various particle volume fractions (0.5–1.5%) at 50–400 W heat inputs in Fig. 10b. The thermal resistance of the heat pipe is reduced by up to 1.0 vol%. Then, it gradually increases with increasing vol% of ZnO nanofluids, owing to NP agglomeration. At all inclination angles of the ZnO nanofluids at lower vol% (0.5–1.0), the total thermal resistance gradually decreases with heat inputs up to 250 W and then varies with heat input increases.

To investigate the heat transfer performance of the dispersant stabilized nanofluids experiments are carried out and compared with and without stabilized ZnO nanofluids and base fluids as represented in Fig. 10c. As a result, the thermal resistance of the ZnO nanofluids is higher than that of dispersant stabilized ZnO nanofluids. Initially, the thermal resistances of heat pipes using base fluids with and without ZnO nanofluids showed high thermal resistance at lower heat inputs and rapidly decreases with corresponding increase in heat inputs from 50 to 400 W^44^. According to Hopkins et al.^44^, this tendency for thermal resistance to vary with heat input is a common feature of heat pipes. Equation (4) is used to calculate the thermal resistance of the heat pipes. Because of the longer hydrophilic AMPS chain length, polymer stabilised nanofluids have higher thermal resistance than ZnO nanofluids, resulting in a decrease in heat transfer.

**Figure 11:**
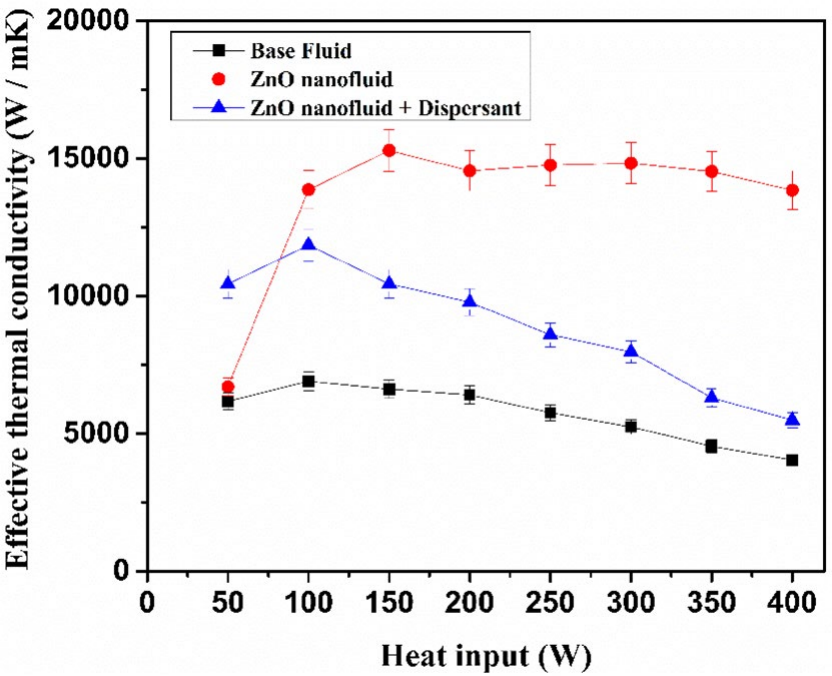
Effective thermal conductivity of nanofluids with respect to heat input.

In the Fig. 11. depicts the thermal conductivity of heat pipe enhancement for various heat inputs. The improvement could be attributed to the deposition of NPs in the wicking mesh. According to the FESEM images, the coating layer on the screen mesh wick may aid in increasing the capillary effect of the wick structure. Surface wettability can be improved by lowering the contact angle and increasing the roughness of the wick surface, which raises the critical heat flux^41^. Recently, Nandy Putra et al.^42^ reported that the primary cause of the improvement in the thermal performance of the heat pipes utilising nanofluids was the creation of a thin coating at the screen mesh wick of the evaporation zone. Since there is less deposition when using a dispersant, the thermal conductivity starts to fall sharply at 100 W. The heat pipe tends to migrate closer to the dry-out situation since its maximum heat transmission capacity is reached at about 100 W. The temperature differential between the evaporator surface and the vapour rises as a result of this partial dry-out situation, which tends to lower the heat transfer coefficient^43,44^. The maximum effective thermal conductivity observed at 38% in 150 W, 29% in 100 W and 11% in 100 W for ZnO nanofluids, Polymer stabilized nanofluids and base fluids respectively.

**Figure 12:**
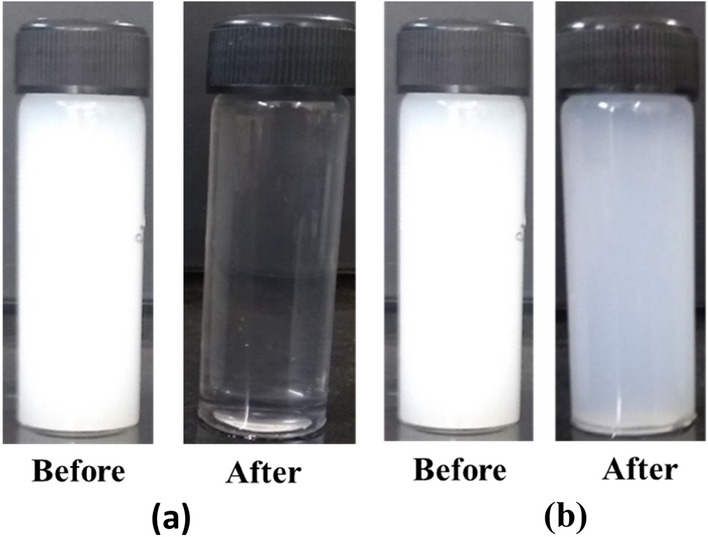
(**a**) Before and after heat pipe experiment of ZnO nanofluids (**b**) Before and after heat pipe experiment of poly (STY-co-AMPS) copolymer stabilized ZnO nanofluids.

Figure 12 shows photographs of ZnO nanofluids with and without dispersants taken before and after the heat pipe experiment. The photography clearly shows that the dispersant-stabilised nanofluids were more stable after the experiment than the ZnO nanofluids.

The thermal resistance at the evaporator is estimated with and without NPs deposition in order to demonstrate the impact of Ps deposition in the wick on heat pipe performance.

17 $$ {\text{R}}_{{\text{e}}} = \frac{{\ln \left( {{{{\text{d}}_{{\text{o, w}}} } \mathord{\left/ {\vphantom {{{\text{d}}_{{\text{o, w}}} } {{\text{d}}_{{\text{i, w}}} }}} \right. \kern-0pt} {{\text{d}}_{{\text{i, w}}} }}} \right)}}{{2\uppi {\text{l}}_{{\text{e}}} {\text{k}}_{{{\text{wall}}}} }} + \frac{{\ln \left( {{{{\text{d}}_{{{\text{o, }}\upomega }} } \mathord{\left/ {\vphantom {{{\text{d}}_{{{\text{o, }}\upomega }} } {{\text{d}}_{{{\text{i, }}\upomega }} }}} \right. \kern-0pt} {{\text{d}}_{{{\text{i, }}\upomega }} }}} \right)}}{{2\uppi {\text{l}}_{{\text{e}}} {\text{k}}_{{{\text{wick}}}} }} $$

where ${\mathrm{R}}_{\mathrm{e}}$—Resistance in evaporator, ${\mathrm{d}}_{\mathrm{o},\mathrm{ w}} , {\mathrm{d}}_{\mathrm{i},\mathrm{ w}}$ are the wall outer and inner diameter, ${\mathrm{d}}_{\mathrm{o},\upomega }, {\mathrm{d}}_{\mathrm{i},\upomega }$ are the wick outer and inner diameter, ${\mathrm{l}}_{\mathrm{e}},$ length of evaporator section, ${\mathrm{k}}_{\mathrm{wall}}$ and ${\mathrm{k}}_{\mathrm{wick}}$ are the thermal conductivity of wall and wick respectiviely.

The combination of a nanofluid and wick has an effective thermal conductivity^44^ estimated as

18 $$ {\text{k}}_{{{\text{wick}}}} = \frac{{{\text{k}}_{1} \left[ {\left( {{\text{k}}_{1} + {\text{k}}_{{\text{s}}} } \right) - \left( {1 - \varphi } \right)\left( {{\text{k}}_{1} - {\text{k}}_{{\text{s}}} } \right)} \right]}}{{\left[ {\left( {{\text{k}}_{1} + {\text{k}}_{{\text{s}}} } \right) + \left( {1 - \varphi } \right)\left( {{\text{k}}_{1} - {\text{k}}_{{\text{s}}} } \right)} \right]}} $$

The following equation is used to determine the porosity of the multiple later of the screen mesh wick ($\varphi$)^28^.

19 $$ \varphi = 1 - \frac{{{\text{n}}\delta_{1} \left( {1 - \upvarphi_{1} } \right)}}{{\delta_{{\text{n}}} }} $$

Then the porosity of the single layer of wick (${\mathrm{\varphi }}_{1})$ is calculated as^45^

20 $$ \upvarphi_{1} = 101 - \frac{{\uppi {\text{SNd}}_{{\text{w}}} }}{4} $$

**Figure 13:**
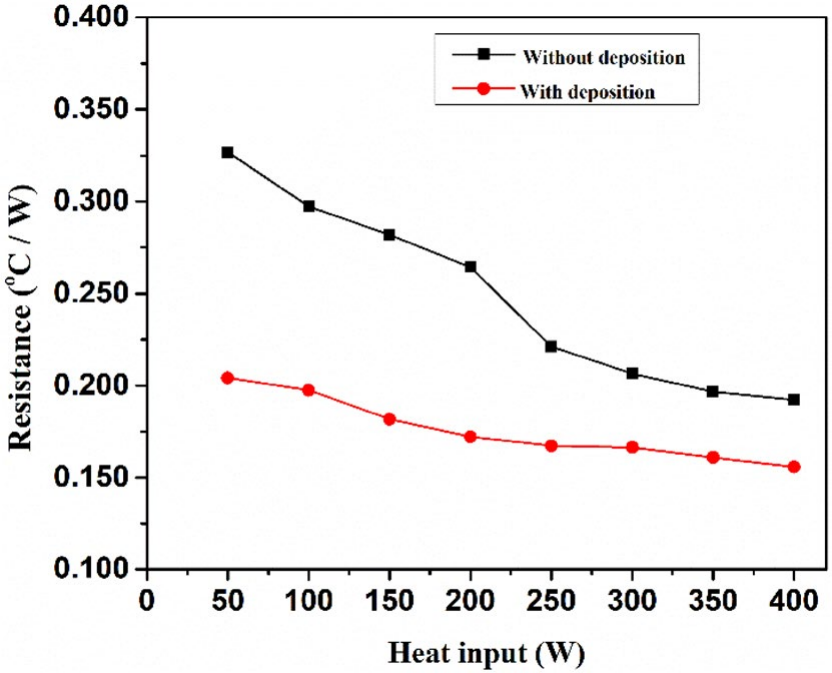
Resistance at the evaporator.

In the Fig. 13. illustrates the measured with and without deposition of NPs at the heat pipe’s evaporator section for varied heat inputs. The evaporator segment of the heat pipe with deposited wick mesh has lower resistance than the corresponding section without deposited wick mesh, it is discovered might be the formation of thin porous layer by the NPs in the fluids.

As a result, heat pipes’ ability to transport heat is reduced. Because of the adsorption of a polymer layer on the surface of the NPs, ZnO nanofluids have lower thermal resistance than dispersant-stabilised nanofluids.

**Figure 14:**
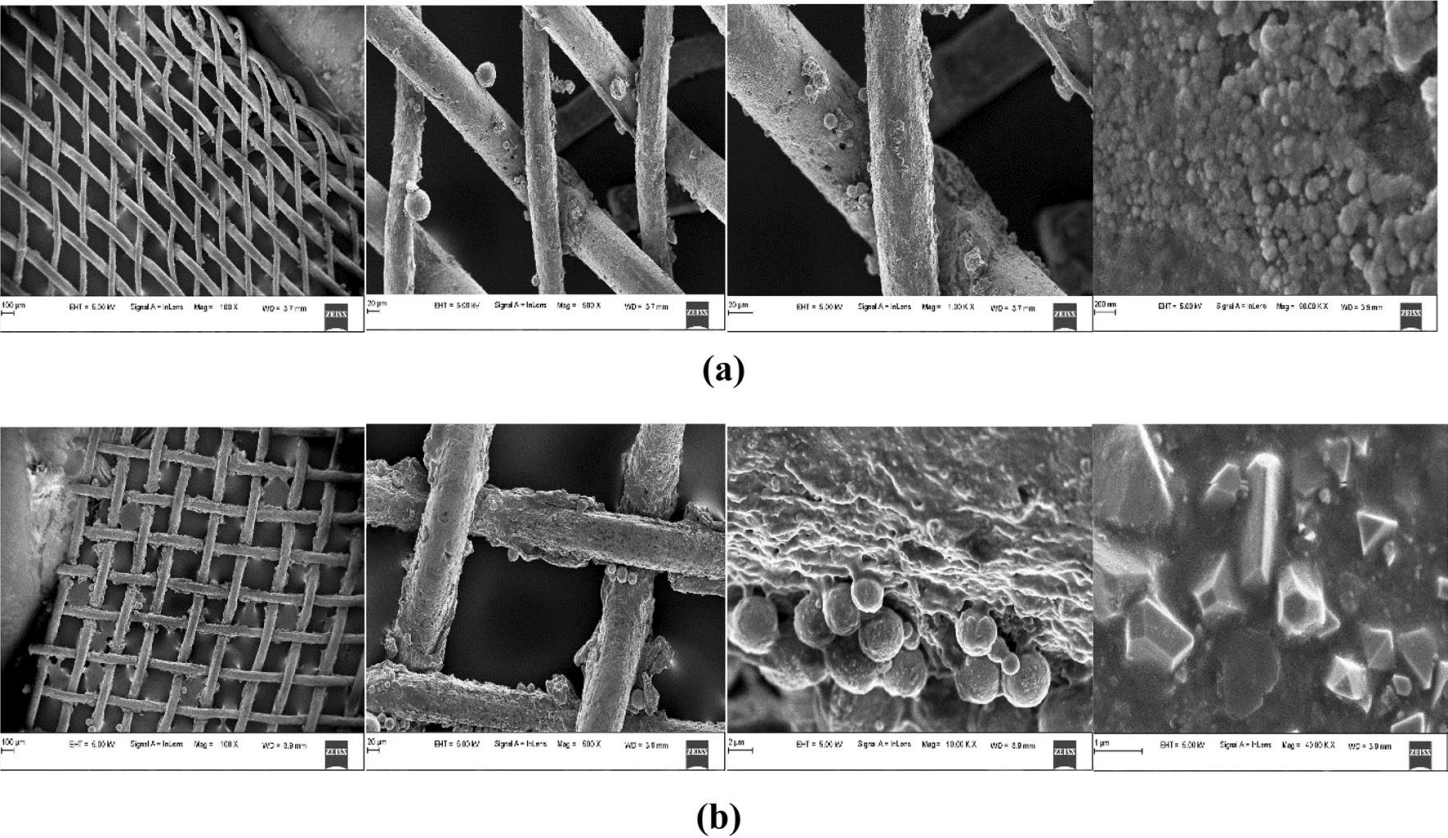
FESEM images for with and without dispersant stabilized ZnO nanofluids (**a**) poly (STY-co-AMPS) copolymer stabilized 1 vol% of ZnO nanofluids (**b**) 1 vol% of ZnO nanofluids after heat pipe experiment.

After all the experiments are completed, heat pipes are cut open and the wick structure was analysed using FESEM shown in Fig. 14. The FESEM images for the ZnO nanofluids stabilized with and without addition of copolymer as dispersant after the heat pipe experiment as shown in Fig. 14a,b. It was found that the NPs are deposited on the wick structure in both cases of nanofluids. However, copolymer stabilised nanofluid showed less deposition than the other one. It clearly indicates that the porous layer on the surface results in more heat transfer. As compared 1 vol% of ZnO nanofluids showed higher heat transfer than 1 vol% of ZnO nanofluids stabilized by 1.5 vol%.

## Conclusion

The thermal conductivities of poly (STY-co-AMPS) copolymer stabilised ZnO nanofluids with a lower concentration of ZnO NPs were experimentally investigated in the current work. In addition, an experiment was performed using the dispersant stabilized nanofluids as working fluids in wick heat pipes in order to measure the thermal resistance at various heat inputs.

1. The structurally designed poly (STY-co-AMPS) copolymer was synthesized by a free radical polymerisation reaction and confirmed by FT-IR, ^1^H-NMR and TGA. ZnO NPs were prepared by microwave irradiation method followed by the preparation of ZnO nanofluids by dispersing different volume fractions of NPs (0.5–1.5 vol%) and dispersant concentration (0.5–2.0 vol%).
2. The optimized concentration of dispersants showed the enhanced thermal performance of the nanofluids. The maximum thermal conductivity 0.931 W/m-K was observed at 1 vol% of ZnO nanofluids stabilized by 1.5 vol% dispersant.
3. The effective thermal conductivity enhancement found to be 38%, 29% and 11% for ZnO nanofluids, polymer stabilized ZnO nanofluids and base fluids respectively.The experimental result was compared with the theoretical models, the H–C model is a good corresponding agreement with the values.
4. Further, the corresponding nanofluids used as working fluid in heat pipe experiments in order to evaluate the thermal resistance of the heat pipe at various temperature distributions is a nonlinear relationship with the volume fraction and dispersant concentration. The maximum uncertainity is found to be 5.41%.
5. The decrease in the thermal resistance of the heat pipe with an increase in the concentration of the dispersant is due to adsorbed layer on the surface of the NPs. The dispersant-stabilized nanofluids exhibited thermal stability after the heat pipe experiment. This confirms that the polymer is stable at high-temperature operation it may be used as a coolant for heat transfer applications.

## Data Availability

All data generated or analysed during this study are included in this published article [and its supplementary information files].

## List of symbols

A: Area [m^2^]
h: Heat of vaporization [J/kg-K]
L: Length [m]
m: Mass flow rate [kg/s]
k: Thermal conductivity [W/m-K]
q: Heat flux [W/m^2^]
Q: Heat transfer rate [W]
R: Resistance [°C/W]
n: Number of the layer of the wick
S: Crimping factor (1.05)
N: Mesh number
T: Temperature [$$^\circ \mathrm{C}]$$
$\Delta T$: Temperature difference between the vapor and condenser
$\Delta t$: Temperature difference between the outlet and inlet of the coolant

## Symbols

$\Phi$: Porosity of multilayer wick
${{\upvarphi }}_{1}$: Porosity of the single layer wick
$$\delta $$: Thickness (m)

## Subscripts

a: Adiabatic
c: Condenser
eff: Effective
e: Evaporator
vap: Vapor
in: Inner
out: Outer
w: Wall
$$\omega $$: Wick
