# Author Correction: Investigation of thermal conductivity and thermal performance of heat pipes by structurally designed copolymer stabilized ZnO nanofluid

**DOI:** 10.1038/s41598-023-43257-w

**Published:** 2023-09-25

**Authors:** K. S. Pavithra, Vinay Parol, A. Brusly Solomon, M. P. Yashoda

**Affiliations:** 1 Department of Chemistry, Research Centre, GM Institute of Technology, Davanagere, 577006 India; 2 Department of Physics, Bapuji Institute of Technology, Davanagere, 577006 India; 3 Micro and Nano Heat Transfer Laboratory, Department of Mechanical Engineering, Centre for Research in Material Science and Thermal Management, Karunya Institute of Technology and Sciences, Coimbatore, India; 4 Department of Chemistry, Manipal Institute of Technology, Manipal Academy of Higher Education, Manipal, Karnataka India 576104

Correction to: *Scientific Reports* 10.1038/s41598-023-39598-1, published online 30 August 2023

The original version of this Article contained an error in Affiliation 4, which was incorrectly given as ‘Department of Chemistry, Manipal Institute of Technology, MAHE, Manipal 576104, India.’

The correct affiliation is listed below:

Department of Chemistry, Manipal Institute of Technology, Manipal Academy of Higher Education, Manipal, Karnataka, India- 576104.

