# Author Correction: Investigation of thermal conductivity and thermal performance of heat pipes by structurally designed copolymer stabilized ZnO nanofluid

**DOI:** 10.1038/s41598-024-52365-0

**Published:** 2024-01-19

**Authors:** K. S. Pavithra, Vinay Parol, A. Brusly Solomon, M. P. Yashoda

**Affiliations:** 1 Department of Chemistry, Research Centre, GM Institute of Technology, Davanagere, 577006 India; 2 Department of Physics, Bapuji Institute of Technology, Davanagere, 577006 India; 3 Micro and Nano Heat Transfer Laboratory, Department of Mechanical Engineering, Centre for Research in Material Science and Thermal Management, Karunya Institute of Technology and Sciences, Coimbatore, India; 4 Department of Chemistry, Manipal Institute of Technology, Manipal Academy of Higher Education, Manipal, 576104 Karnataka India

Correction to: *Scientific Reports* 10.1038/s41598-023-39598-1, published online 30 August 2023

In the original version of this Article, K. S. Pavithra was incorrectly listed as a corresponding author. The correct corresponding author for this Article is M. P. Yashoda. Correspondence and request for materials should be addressed to yashoda.mp@manipal.edu.

The original Article has been corrected.

